# Lower SARS-CoV-2 Seroprevalence among Cancer Patients in Sub-Saharan Africa

**DOI:** 10.3390/jcm11154428

**Published:** 2022-07-29

**Authors:** For Yue Tso, Salum J. Lidenge, John R. Ngowi, Phoebe B. Peña, Ashley A. Clegg, Owen Ngalamika, Chacha J. Mwita, Julius Mwaiselage, Charles Wood

**Affiliations:** 1 Department of Interdisciplinary Oncology, and the Stanley S Scott Cancer Center, Louisiana State University Health Sciences Center, New Orleans, LA 70112, USA; ftso@lsuhsc.edu; 2 Ocean Road Cancer Institute, Dar es Salaam P.O. Box 3592, Tanzania; sjlidenge@yahoo.co.uk (S.J.L.); jrngowi146@gmail.com (J.R.N.); cjosiahm@gmail.com (C.J.M.); jmwaiselage@yahoo.com (J.M.); 3 Department of Clinical Oncology, Muhimbili University of Health and Allied Sciences, Dar es Salaam P.O. Box 65001, Tanzania; 4 Nebraska Center for Virology, Lincoln, NE 68583, USA; ppena@huskers.unl.edu (P.B.P.); ashleyaclegg@gmail.com (A.A.C.); 5 School of Biological Sciences, University of Nebraska-Lincoln, Lincoln, NE 68588, USA; 6 Dermatology and Venereology Section, University Teaching Hospitals, University of Zambia School of Medicine, Lusaka P.O. Box 50110, Zambia; owen_ngalamika@yahoo.com

**Keywords:** cancer, SARS-CoV-2, COVID-19, seroprevalence, sub-Saharan Africa, tuberculosis

## Abstract

Background: Despite the high COVID-19 morbidity and mortality rates across the world, the reported rates in sub-Saharan Africa (SSA), which has a higher burden of other infectious diseases and overwhelmed healthcare systems, remain relatively low. This study aims to better understand the potential factors that contribute to this phenomenon, especially among cancer patients who are considered as a high-risk group for developing severe COVID-19. Methods: Plasma samples collected during the COVID-19 pandemic from SARS-CoV-2 unvaccinated cancer and potential blood donor populations were analyzed for SARS-CoV-2 (spike and nucleocapsid proteins) antibodies by an immunofluorescence assay. The relationships between SARS-CoV-2 seroprevalences and study variables were determined using a logistic regression analysis. Results: High seroprevalence against the SARS-CoV-2 spike and nucleocapsid proteins were found among the SARS-CoV-2 unvaccinated COVID-19 pandemic populations in SSA. However, the cancer patients demonstrated a lower seroprevalence compared to potential blood donors. There was also an association between mild COVID-19 symptoms with prior tuberculosis vaccination among cancer patients. Conclusion: Cancer patients in SSA tend to have a relatively lower SARS-CoV-2 seroprevalence compared to potential blood donors recruited from the same geographic locations during the COVID-19 pandemic. More study is required to determine its cause and potential impact on SARS-CoV-2 vaccination among cancer patients.

## 1. Introduction

Since its discovery in late 2019, severe acute respiratory syndrome coronavirus 2 (SARS-CoV-2) infection has rapidly spread all over the world and caused the ongoing coronavirus disease 2019 (COVID-19) pandemic [1]. At the time of writing, over 557 million people had been infected worldwide and more than 6 million had lost their lives due to COVID-19 [2]. A majority of the countries that reported high COVID-19 morbidity and mortality are in North and South America, Europe, and Asia, especially in developed countries such as the United States. The higher risk for more severe COVID-19 disease, hospitalization, and mortality rates have been associated with pre-existing conditions such as obesity, diabetes, and active cancer [3]. Although these conditions also exist in sub-Saharan Africa (SSA), the number of COVID-19 cases and deaths in SSA is surprisingly low compared to other countries worldwide [2]. Moreover, SSA countries also have high HIV-1 prevalence, HIV-associated cancers, and other infectious disease burdens such as tuberculosis, malaria, and parasitic diseases that exert a significant strain on the healthcare systems [4,5]. Importantly, both HIV-1 and cancer have been reported to be co-morbidities associated with severe COVID-19 disease [6,7]. HIV-1 infection can severely damage and reduce the ability of the immune system to respond to infection such as SARS-CoV-2, thereby rendering HIV-1-infected individuals more susceptible to SARS-CoV-2 infection and severe COVID-19 disease [8,9]. Similarly, it was also expected that perhaps the dysfunctional immune responses in cancer patients would render them more susceptible to SARS-CoV-2 infection, leading to high COVID-19 mortality rates [10]. Recent studies conducted in Japan and Germany suggest that SARS-CoV-2 seroprevalence was lower among cancer patients [11,12]. However, whether lower SARS-CoV-2 seroprevalence also holds true in the SSA cancer patient population, especially in the presence of HIV-1 infection, is not clear, although no increase in COVID-19 cases and deaths among cancer patients has been reported from SSA [13].

Several potential explanations for low COVID-19 morbidity and mortality in Africa compared to the rest of the world have been proposed. The predominant argument is that Africa has a relatively younger population as compared to that in America or Europe, which, therefore, limited the spread of SARS-CoV-2 and severity of COVID-19 [14]. However, similar to the rest of the world, cancer in Africa tends to mostly occur among the elderly as cancer incidence increases with age [15]. Additionally, countries in SSA did not apply strict or prolonged COVID-19 restriction measures such as those enacted in developed countries, which can limit SARS-CoV-2 transmission among the populations [16]. Access to or willingness to accept the SARS-CoV-2 vaccines has been low, as only 12% and 5% of populations in Zambia and Tanzania, respectively, are vaccinated against SARS-CoV-2 [2]. Together, these facts suggest that, for some unknown factors, SSA populations could be more resistant to the development of severe COVID-19.

An alternative hypothesis for the relatively fewer COVID-19 cases and deaths in SSA is that prior exposure to other strains of human coronaviruses (HCoV), such as NL63, may have elicited cross-reactive immune responses against SARS-CoV-2 in SSA populations. This hypothesis is possible as HCoV-NL63 is the only known HCoV that utilizes the same receptor, angiotensin-converting enzyme 2 (ACE2), as SARS-CoV-2 [17]. Our previous data supported this hypothesis by demonstrating the presence of pre-existing cross-reactive humoral response against SARS-CoV-2 in pre-pandemic SSA populations [18]. Other groups have since demonstrated the presence of pre-existing cross-reactive T-cell immune response against SARS-CoV-2 [19]. Hence, it is possible that cancer patients in SSA may also be partially protected from severe COVID-19 because of prior exposure to other HCoVs. However, whether cancer patients in SSA still have a higher risk for SARS-CoV-2 infection and for severe COVID-19 disease compared to the potential blood donor population has not been investigated.

Studies from developed countries have shown conflicting results regarding seroprevalence of SARS-CoV-2 among cancer patients. A study in France reported similar SARS-CoV-2 seroprevalence between cancer patients and healthcare workers, whereas another study from Spain reported higher SARS-CoV-2 seroprevalence among cancer patients [20,21]. Moreover, several studies from Japan, Germany, and Italy reported lower seroprevalence of SARS-CoV-2 among cancer patients [11,12,22]. There are no studies on the seroprevalence of SARS-CoV-2 among cancer patients from SSA.

To investigate whether cancer patients in SSA may be more or less susceptible to SARS-CoV-2 infection, we determined the seroprevalence of SARS-CoV-2 in unvaccinated cancer and potential blood donor populations recruited during the COVID-19 pandemic, and analyzed other study variables such as HIV-1 co-infection and COVID-19-related symptoms to determine whether there are co-factors that could affect their susceptibility to infection and disease. Although the seroprevalence of anti-SARS-CoV-2 antibodies in some African countries have been reported by several studies, these studies only focused on the general population [23,24,25]. Our study is unique as we focused on the SSA cancer population, which is consider at high-risk of developing severe COVID-19. In addition, our study utilized pre-pandemic archived samples to confirm the presence and prevalence of cross-reactivity against SARS-CoV-2 in cancer patients in comparison to potential blood donors.

## 2. Materials and Methods

### 2.1. Study Design, Subjects, and Samples

This was a cross-sectional study comprising 479 cancer and 899 potential blood donors who were consenting subjects, ≥18 years of age, and of both genders from Dar es Salaam, Tanzania and Lusaka, Zambia, collected during the COVID-19 pandemic between February and November 2021, or between 14 months and 23 months into the pandemic since the discovery of SARS-CoV-2 in December 2019. The potential blood donors were recruited among voluntary prospective blood donors in Zambia and Tanzania who were not known to have any cancer. The cancer patients were recruited during their initial visit, just after cancer diagnosis and prior to any cancer treatment, from either the University Teaching Hospital in Zambia or Ocean Road Cancer Institute in Tanzania. All cancers in this study were diagnosed by histopathological examination of the biopsied tissues according to the standard national guidelines for diagnosis of specific cancers in the respective countries. After consent, each study participant provided a plasma sample and answered a short questionnaire on sociodemographic information, such as age, gender, and medical history including HIV status, all TB and COVID-19 vaccinations if any, and COVID-19 symptoms (loss of smell/taste, difficulty breathing, fever, cough, and sore throat). HIV status was also confirmed through a serology test as stated below. Pre-pandemic cohorts consisting of archived plasma samples from 373 cancer and 900 potential blood donors, ≥18 years of age, and of both genders from Dar es Salaam, Tanzania and Lusaka, Zambia, collected prior to the COVID-19 pandemic (between March 2015 to October 2019) from the same local facilities as the pandemic cohort, were also evaluated for comparison. The study was approved by the institutional review boards from the University of Zambia Biomedical Research Ethics Committee, the Tanzania National Institute for Medical Research, Ocean Road Cancer Institute, University of Nebraska–Lincoln, and Louisiana State University Health Sciences Center New Orleans.

### 2.2. HIV-1 Serological Testing

HIV-1 serological status was determined using either the Alere Determine HIV-1/2 Ag/Ab Combo test in Zambia or the HIV Rapid Test Algorithm in Tanzania. The HIV-1 serological results were confirmed in our lab in the USA using the HIV-1-2.0 First Response kit (Premier Medical Corporation Limited, Daman, India).

### 2.3. Immunofluorescence Assay against SARS-CoV-2 Spike and Nucleocapsid Proteins

To detect SARS-CoV-2 seroprevalence in the plasma samples, an immunofluorescence assay (IFA) against the SARS-CoV-2 spike and nucleocapsid proteins was used as previously described [18]. Briefly, transfected HEK-293T cells (ATCC, Manassas, VA, USA) expressing either the spike or nucleocapsid proteins of SARS-CoV-2 (Addgene, Watertown, MA, USA and Sino Biological, Chesterbrook, PA, USA, respectively) were fixed and seeded onto 12-well polytetrafluoroethylene (PTFE)-printed slides (Electron Microscopy Sciences, Hatfield, PA, USA). The plasma samples were diluted 1:20 in 1× PBS with 0.1% of Tween 20, and 15 µL of the diluted plasma sample was added to each corresponding well on the slide and incubated for 1 h at 37 °C. After washing, the slides were incubated with secondary mouse monoclonal anti-human IgG antibody (ATCC, Manassas, VA, USA), followed by the removal of unbound antibodies and incubation with tertiary CY2-conjugated donkey anti-mouse IgG (Jackson ImmunoResearch Laboratories, West Grove, PA, USA). The cells were then counterstained with Evans blue solution. The stained IFA was evaluated by three independent readers using a Nikon Eclipse 50i fluorescence microscope to determine positive or negative signals on the slide, and only concordant results from at least two independent readers were reported as the outcome.

### 2.4. Statistical Analysis

Categorical variables were described using frequencies and percentages. Non-normally distributed continuous variables were described using median and range. The comparison between non-normally distributed continuous variables were analyzed using the non-parametric Mann–Whitney test. The association between categorical variables were analyzed using chi-square tests. The differences in the proportion of SARS-CoV-2 (overall and spike/nucleocapsid) humoral responses between cancer and potential blood donors were calculated. The 95% confidence intervals of the proportional differences were included. Univariate and multiple logistic regressions were used to investigate the relationship between covariates (age, sex, HIV status, tuberculosis (TB) vaccination, COVID-19-related symptoms, and household size) and SARS-CoV-2 seroprevalence in pandemic cancer and potential blood donors. Additionally, multiple logistic regression was used to model the relationship between several study variables and the presence of COVID-19-related symptoms in the SARS-CoV-2 seropositive cancer patient and potential blood donor pandemic populations. Data were analyzed using GraphPad (GraphPad Software, San Diego, CA, USA) and SAS version 9.4 software (SAS Institute, Cary, NC, USA).

## 3. Results

### 3.1. Characteristics of the Study Cohort

The pandemic cancer population (N = 479) was significantly older than the potential blood donors (N = 899), with a median age of 49 years old compared to 33 years old (Mann–Whitney U = 450425.5, *p* < 0.0001, r = 0.46), respectively (Table 1). The cancer diagnosis comprised, predominantly, the most common forms of malignancies seen at the participating cancer centers in SSA, such as Kaposi’s sarcoma, cervical, and breast cancers, but it also included a wide range of other cancers such as lung, colon, prostate, and esophagus cancers, as well as melanoma and lymphoma. The majority of the cancer participants were females (72.6%), whereas 60.5% of potential blood donors were males. The commonest cancers in our pandemic population were cervical cancer (n = 204, 42.95%), breast cancer (n = 63, 13.26%), and Kaposi’s sarcoma (n = 38, 8%) (Supplementary Table S1). The HIV prevalence was significantly higher among the cancer population than potential blood donors, at 31.3% versus 6.7% (*p* < 0.0001), respectively. Cancer individuals also reported having more household members than potential blood donors, with a median of 5 members compared to 4 members (Mann–Whitney U = 390187.5, *p* < 0.0001, r = 0.25), respectively. There was an association between having cancer and the presence of COVID-19-related signs/symptoms such as loss of smell/taste, difficulty breathing, fever, cough, and sore throat (*p* < 0.0001). Specifically, 27.3% of cancer patients reported having COVID-19-related signs/symptoms, whereas 6.9% of potential blood donors reported having COVID-19-related signs/symptoms. Despite the reported COVID-19-related signs/symptoms, none of the cancer and potential blood donor study participants required hospitalization due to severe COVID-19 disease and all were unvaccinated against SARS-CoV-2. For comparison of SARS-CoV-2 seroprevalence prior to the pandemic, 373 cancer and 900 potential blood donor participants’ archived pre-pandemic (before December 2019) plasma samples were collected from identical sites in SSA. The pre-pandemic cancer patients were older compared to potential blood donors, with a median age of 39 versus 31 years (Mann–Whitney U = 309152.5, *p* < 0.0001, r = 0.34).

### 3.2. Seroprevalence against SARS-CoV-2 Spike and Nucleocapsid Proteins 

**Figure 1:**
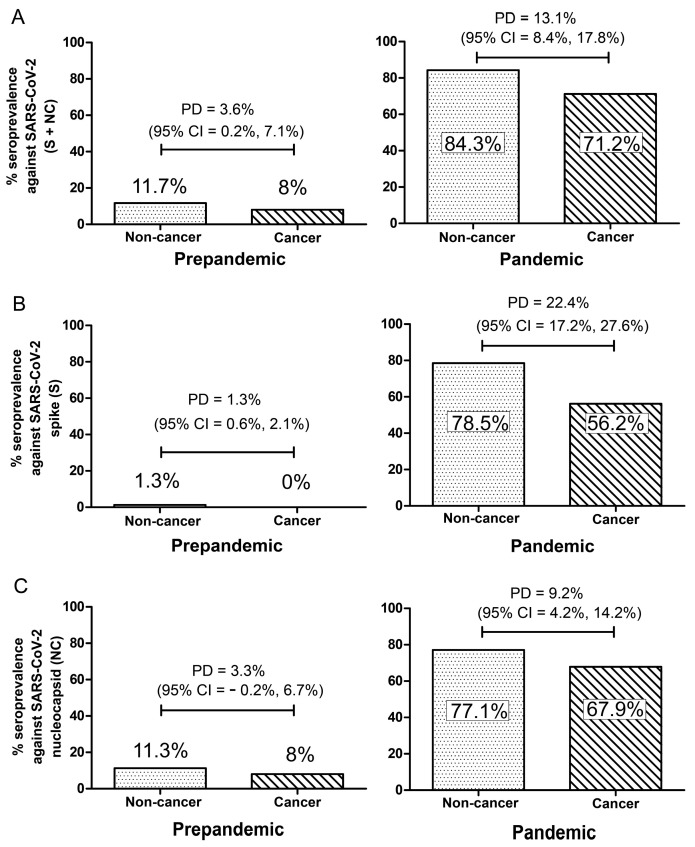
Seroprevalence against SARS-CoV-2 spike (S) and nucleocapsid (NC) in SSA pandemic plasma samples (right), with pre-pandemic archived samples for comparison (left). (**A**) S + NC. (**B**) S alone. (**C**) NC alone. “PD” represents proportional difference between the groups. “CI” represents confidence intervals.

Among the pandemic populations, the proportion of individuals who were SARS-CoV-2 seropositive was 84.3% in potential blood donors versus 71.2% in cancer patients, with a proportional difference of 13.1 % between the groups (95% CI = 8.4%, 17.9%) (Figure 1A). On separating the humoral SARS-CoV-2 response into spike and nucleocapsid proteins responses, the cancer patients still had lower seroprevalence compared to potential blood donors. The proportion of individuals who were seropositive against the SARS-CoV-2 spike protein was 78.5% in potential blood donors versus 56.2% in cancer patients, with a proportional difference of 22.4% (95% CI = 17.2%, 27.6%) (Figure 1B). Likewise, the proportion of individuals who were seropositive against the SARS-CoV-2 nucleocapsid protein was 77.1% in potential blood donors versus 67.9% in cancer patients, with a proportional difference of 9.2% (95% CI = 4.2%, 14.2%) (Figure 1C). Similarly, after adjusting for age, sex, HIV status, household size, and BCG vaccination history, the probability of SARS-CoV-2 seropositivity was lower in cancer patients compared to potential blood donors (total seropositivity: OR = 0.535, 95% CI = 0.380–0.753, *p*-value = 0.0003; spike protein: OR = 0.431, 95% CI = 0.318–0.585, *p*-value = 0.0001; Nucleocapsid protein: OR = 0.721, 95% CI = 0.526–0.989, *p*-value = 0.042) (Supplementary Table S2).

For the pre-pandemic populations, the proportion of individuals who were SARS-CoV-2 seropositive was 11.7% in potential blood donors versus 8% in cancer, with a proportional difference of 3.6% (95% CI = 0.2%, 7.1%) (Figure 1A). On separating the humoral SARS-CoV-2 response into spike and nucleocapsid protein responses, cancer patients also had a lower seroprevalence against the SARS-CoV-2 spike protein. Specifically, the proportion of individuals who were seropositive against the SARS-CoV-2 spike protein was 1.3% in potential blood donors versus 0% in cancer patients, with a proportional difference of 1.3% (95% CI = 0.6%, 2.1%) (Figure 1B). However, there was not a statistically significant difference in the proportion of individuals who were seropositive against the SARS-CoV-2 nucleocapsid protein between potential blood donors and cancer patients (11.3% versus 8%, proportional difference = 3.3%, 95% CI = −0.2%, 6.7%) (Figure 1C). After adjusting for age, sex, and HIV status, the probability of seropositivity was similar between cancer and potential blood donors (total seropositivity: OR = 0.518, 95% CI = 0.255–1.052, *p*-value = 0.069; spike protein: OR = 0.168, 95% CI = 0.0009–3.308, *p*-value = 0.241; Nucleocapsid protein: OR = 0.541, 95% CI = 0.265–1.102, *p*-value = 0.091) (Supplementary Table S3).

### 3.3. Logistic Regression Analysis

To investigate the relationship between study variables including age, sex, HIV status, TB vaccination, COVID-19-related symptoms, household size, and SARS-CoV-2 seroprevalence in the cancer patients and potential blood donors during the pandemic, univariate and multivariate logistic regression analyses were performed. Cancer patients were classified into five major groups; breast cancer, cervical cancer, Kaposi’s sarcoma, hematological malignancies, and other solid malignancies. In the analysis of this cohort of pandemic cancer patients, cancer type was the only variable associated with SARS-CoV-2 seroprevalence in the univariate analysis. Specifically, the probability of seropositivity was higher among breast cancer patients (OR = 2.379, 95% CI = 1.012–5.592, *p*-value = 0.047) and cervical cancer patients (OR = 2.249, 95% CI = 1.105–4.580, *p*-value = 0.026) compared to Kaposi’s sarcoma patients. However, the probability of seropositivity was similar to Kaposi’s sarcoma among all cancer categories in a multivariable logistic regression (Table 2). Therefore, all cancer types were combined for subsequent analyses. We did not find any association between SARS-CoV-2 seroprevalence and all study variables among the potential blood donor pandemic (Table 3) populations using univariate and multivariate analyses (*p* > 0.05). However, the analysis of the relationship between study variables and presence of COVID-19-related symptoms showed that HIV-negative individuals were found to have lower odds of having COVID-19-related symptoms in the potential blood donors in both univariate (OR = 0.383, 95% CI = 0.179–0.819, *p* = 0.0134) and multivariate analyses (OR = 0.389, 95% CI = 0.176–0.858, *p* = 0.0194) (Table 4), but not in the cancer population (Table 5). Interestingly, analysis of the cancer population showed that individuals who previously received a TB vaccination were more likely to report having COVID-19-related symptoms in both univariate (OR = 2.602, 95% CI = 1.198–5.652, *p* = 0.0157) and multivariate analyses (OR = 2.511, 95% CI = 1.114–5.661, *p* = 0.0264) (Table 5). Additional analysis that focused on SARS-CoV-2 seropositive potential blood donors only showed that sex (OR = 2.571, 95% CI = 1.452–4.551, *p* = 0.0012), HIV status (OR = 0.295, 95% CI = 0.134–0.646, *p* = 0.0023), and history of TB vaccination (OR = 0.441, 95% CI = 0.212–0.918, *p* = 0.0287) were significantly associated with the presence of COVID-19-related symptoms in univariate analysis (Table 6). However, in multivariate analysis, only being female (OR = 1.950, 95% CI = 1.062–3.581, *p* = 0.0312) and HIV status (OR = 0.305, 95% CI = 0.134–0.694, *p* = 0.0047) remained significantly associated with the presence of COVID-19-related symptoms. There was a marginally significant relationship between having COVID-19-related symptoms and those who reported a history of TB vaccination (OR = 0.482, 95% CI = 0.223–1.042, *p* = 0.0635) (Table 6). A similar analysis of SARS-CoV-2 seropositive cancer individuals showed that only the age was significantly associated with the presence of COVID-19-related symptoms in both univariate (OR = 1.026, 95% CI = 1.007–1.045, *p* = 0.006) and multivariate analysis (OR = 1.029, 95% CI = 1.010–1.049, *p* = 0.0034) (Table 7).

## 4. Discussion

In this cross-sectional study, we compared the seroprevalence of SARS-CoV-2 among cancer patients and potential blood donors recruited in SSA during the COVID-19 pandemic. We found that the cohort of cancer patients from SSA tends to have a relatively lower SARS-CoV-2 seroprevalence compared to the potential blood donors recruited from the same geographic locations. Despite having a high prevalence of HIV-1 infection, HIV-associated cancers, and other COVID-19-associated co-morbidities, the number of COVID-19 cases and deaths remains low in SSA compared to developed countries in this third year of the COVID-19 pandemic [2]. Previous studies have shown the presence of pre-existing SARS-CoV-2 cross-reactive humoral and cellular responses in Africa, which might explain this partial protection against severe COVID-19 disease in the general SSA populations [18,19]. The rationale is that other human coronaviruses (HCoVs) such as HCoV-OC43, HCoV-HKU-1, HCoV-NL63, and HCoV-229E, which cause the common cold, may elicit cross-reactive humoral and cellular responses against SARS-CoV-2 due to their close genome similarity, especially within the nucleocapsid. Distribution of these HCoVs varies throughout the world and could be seasonal. It is possible that the SSA population was exposed to some of these HCoVs prior to the COVID-19 pandemic and these infections, in turn, offered partial protection against severe COVID-19 diseases by eliciting cross-reactive humoral and cellular responses against SARS-CoV-2 viral proteins. It is intriguing that potential blood donor SARS-CoV-2 seropositive females tend to be more likely to report COVID-19-related symptoms. It is possible that women are more conscientious about their health than men, and hence, more aware of the presence of COVID-19-related symptoms. A recent study of a Finnish cohort also reported a similar finding [26].

In this study, we showed that this partial protection may be extended to cancer patients in SSA during the current pandemic, despite cancer being a risk factor for severe COVID-19 based on studies from developed countries [27]. This is supported by the fact that none of our study participants, from both the cancer and potential blood donor cohorts, developed severe COVID-19 disease that required hospitalization in the absence of SARS-CoV-2 vaccination. Additionally, the high SARS-CoV-2 seroprevalence among the unvaccinated cancer patients and potential blood donors in SSA is near the theoretical threshold of herd immunity, which further supports the finding of relatively lower COVID-19 hospitalizations and deaths in SSA [28]. The high SARS-CoV-2 seroprevalence also suggests that SARS-CoV-2 infection may be more widespread among the SSA populations than realized, with the infected individuals either displaying mild COVID-19 signs/symptoms or completely asymptomatic. However, whether this high SARS-CoV-2 seroprevalence translates into protection against new emerging SARS-CoV-2 variants, such as the Delta and Omicron strains, is not known.

It is possible that the relatively lower SARS-CoV-2 seroprevalence and more frequent mild symptoms reported in cancer patients could be due to increased awareness of their well-being or social behavioral changes after the onset of cancer-related illness that limited their exposure to SARS-CoV-2. For example, as cancer develops, the patients may experience fever, pain, feeling tired, or other discomforts that render them more likely to rest at home rather than go out with friends or family. Other cancers such as Kaposi’s sarcoma that form numerous skin lesions may also make the patients choose to stay home to avoid social stigma due to the unsightly skin lesions/tumors. By choosing to stay home or avoid social functions, these cancer patients may have limited their exposure to SARS-CoV-2. Alternatively, cancer individuals might mount an attenuated immune response upon infection that could have prevented the development of a robust SARS-CoV-2 humoral response, perhaps at a level below the detection level of our assay. This is supported by a recent study where the SARS-CoV-2 spike and nucleocapsid-specific IgG level was lower among cancer patients [12]. This tempered immune response in cancer patients may have prevented the cytokine storm that could have led to severe COVID-19 disease [29]. On the other hand, this attenuated humoral response of cancer patients may also potentially impact the effectiveness of SARS-CoV-2 vaccines in these individuals. Further studies will be needed to investigate these possibilities.

Analysis of the study cohort during the pandemic revealed significant differences in age, sex, and HIV status between the cancer patient and potential blood donor pandemic populations. However, these differences are unrelated to COVID-19 since they are also present in the pre-pandemic cohort and are likely to relate to the local social-economic and environmental factors. The cancer population had higher HIV prevalence compared to potential blood donors. This was expected since HIV-associated malignancies such as Kaposi’s sarcoma and cervical cancer are frequent in SSA, whereas the HIV prevalence in the potential blood donors reflected the general population prevalence [30]. The majority of the cancer population were females due to cervical and breast cancers being the most common cancers in the region. Conversely, the high percentage of male participants in the potential blood donors is likely due to local cultural habits where mostly men volunteer to donate blood [31]. Intriguingly, we did find that cancer patients who had received a TB vaccination were more likely to report COVID-19-related symptoms. This observation is contrary to studies in the USA and Europe that reported lower SARS-CoV-2 seroprevalence and COVID-19-related symptoms in the tuberculosis-vaccinated potential blood donors and medical professionals [32,33]. This difference from other studies is possibly due to the low number of TB unvaccinated individuals in our study cohort. Therefore, a larger sample size including TB unvaccinated individuals with cancer will be needed to confirm our observation in the future. Although highlighting important associations among cancer and potential blood donors exposed to SARS-CoV-2 in SSA, our study did not find any significant association between SARS-CoV-2 seroprevalence and COVID-19-related symptoms or with other study variables. This illustrates the distinctiveness of the humoral response in each infected individual and the decoupling between the humoral response and COVID-19-related symptoms, suggesting that other components of the immune system, such as cellular immunity, might have a more prominent role in the onset or suppression of COVID-19-related symptoms.

Although our COVID-19-related signs/symptoms are based on CDC recommendations, we cannot exclude the possibility that some of the COVID-19-related signs/symptoms reported by some of the study subjects were caused by other infections such as the common cold. It is also possible that our study may have missed some individuals who were recently infected with SARS-CoV-2, as it generally takes two weeks to mount the SARS-CoV-2-specific humoral response [34], as well as individuals whose SARS-CoV-2 humoral response may have decayed over time. Although our SARS-CoV-2 immunofluorescence assay can detect a cross-reactive response, as demonstrated by the low response (~8 to 11%) against the SARS-CoV-2 nucleocapsid and even lower response (~0 to 1%) against the SARS-CoV-2 spike in the pre-pandemic samples, the responses from pandemic samples are far higher at 67 to 77% for the nucleocapsid and 56 to 78% for the spike. Therefore, cross-reactivity detection played a minimal role in the results of pandemic seroprevalence. Additionally, this was a prospective cross-sectional study with participants recruited from local blood donation and cancer centers, and we had no nasopharyngeal swab samples for RT-PCR confirmation of SARS-CoV-2 infection. Regardless, RT-PCR detection of SARS-CoV-2 only documents acute or prolonged infection but not past asymptomatic SARS-CoV-2 infections, and therefore, is not as informative for this seroepidemiology study. Furthermore, as a result of differences in distribution of variables such as age, gender, and HIV status between the pre-pandemic and pandemic groups in this study, this limited our direct comparison of the two groups and, hence, the pre-pandemic group was used as a reference to show presence and prevalence of cross-reactive anti-SARS-CoV-2 responses in the study population. Nevertheless, the relatively lower SARS-CoV-2 seroprevalence among SSA cancer patients may indicate an attenuated immune system that prevented a robust SARS-CoV-2-specific humoral response. Further studies on the impact that this tempered immune response may have on SARS-CoV-2 vaccines and COVID-19 in SSA cancer patients are warranted.

## Figures and Tables

**Table 1:** Study cohort characteristics. Non-parametric Mann–Whitney test of non-normally distributed continuous variables.

Pre-Pandemic	Potential Blood Donors	Cancer	*p*-Value
Sample size, N	900	373	
Age, median (IQR) years	31 (25–39)	39 (33–45)	<0.0001
Male, N (%)	710 (78.9%)	155 (41.6%)	<0.0001
HIV+, N (%)	29 (3.2%)	282 (75.6%)	<0.0001
**Pandemic**	**Potential Blood Donors**	**Cancer**	***p*-Value**
Sample size, N	899	479	
Age, median (IQR) years	33 (26–43)	49 (40–60)	<0.0001
Male, N (%)	544 (60.5%)	131 (27.4%)	<0.0001
HIV+, N (%)	60 (6.7%)	150 (31.3%)	<0.0001
Number of household members, median (IQR) *Samples with missing information, N*	4 (2–5) *22*	5 (3–7) *2*	<0.0001
Received TB vaccination, N (%) *Samples with missing information, N*	800 (89.5%) *5*	418 (87.8%) *3*	0.3488
Have COVID-related symptoms, N (%) *Samples with missing information, N*	62 (6.9%) *1*	130 (27.3%) *3*	<0.0001

Note: TB denotes tuberculosis; N denotes sample size.

**Table 2:** Logistic regression analysis of factors associated with SARS-CoV-2 seroprevalence within the **pandemic cancer** populations.

Cancer Population	Unadjusted Analysis	*p*-Value	Adjusted Analysis	*p*-Value
***Cancer type***				
Breast cancer	2.379 (1.012–5.592)	0.047	2.023 (0.705–5.801)	0.19
Cervical cancer	2.249 (1.105–4.580)	0.026	1.984 (0.783–5.029)	0.149
Hematological malignancies	1.133 (0.305–4.216)	0.852	0.985 (0.255–3.805)	0.982
Other solid malignancies	2.042 (0.989–4.216)	0.054	1.885 (0.814–4.362)	0.139
Kaposi’s sarcoma	ref		ref	
***Age***	0.997 (0.983–1.011)	0.653	0.992(0.977–1.007)	0.29
***Sex***				
Female	1.257 (0.965–1.637)	0.2346	1.121 (0.615–2.043)	0.7089
Male	ref		ref	
***HIV status***				
Negative	1.193 (0.783–1.818)	0.4106	1.1 (0.672–1.801)	0.705
Positive	ref		ref	
***Received TB vaccination***				
Yes	1.358 (0.917–2.013)	0.3074	1.401 (0.767–2.558)	0.2731
No	ref		ref	
***COVID-19-related symptoms***				
Yes	0.755 (0.488–1.166)	0.2052	0.726 (0.465–1.135)	0.1604
No	ref		ref	
***Household size***	0.992 (0.924–1.064)	0.8181	0.984 (0.915–1.059)	0.6702

Note: TB denotes tuberculosis.

**Table 3:** Logistic regression analysis of factors associated with SARS-CoV-2 seroprevalence within the **pandemic potential blood donors**.

Potential Blood Donors Population	Unadjusted Analysis OR (95% CI)	*p*-Value	Adjusted Analysis OR (95% CI)	*p*-Value
***Age***	1.002 (0.988–1.017)	0.7428	1.003 (0.987–1.018)	0.7309
***Sex***				
Female	0.860 (0.597–1.238)	0.4177	0.946 (0.642–1.393)	0.7779
Male	ref		ref	
***HIV status***				
Negative	1.224 (0.620–2.417)	0.5596	1.268 (0.627–2.562)	0.5088
Positive	ref		ref	
***Received TB vaccination***				
Yes	1.304 (0.753–2.256)	0.3432	1.210 (0.683–2.143)	0.5141
No	ref		ref	
***COVID-19-related symptoms***				
Yes	1.104 (0.532–2.293)	0.7904	1.161 (0.551–2.444)	0.6946
No	ref		ref	
***Household size***	0.954 (0.878–1.036)	0.2609	0.972 (0.891–1.061)	0.5282

Note: TB denotes tuberculosis.

**Table 4:** Logistic regression analysis of factors associated with COVID-19-related symptoms within the **pandemic potential blood donors**.

Potential Blood Donors Population	Unadjusted Analysis OR (95% CI)	*p*-Value	Adjusted Analysis OR (95% CI)	*p*-Value
***Age***	1.016 (0.997–1.036)	0.1074	1.007 (0.987–1.029)	0.4798
***Sex***				
Female	1.958 (1.165–3.291)	0.0112	1.510 (0.870–2.624)	0.1433
Male	ref		ref	
***HIV status***				
Negative	**0.383 (0.179–0.819)**	**0.0134**	**0.389 (0.176–0.858)**	**0.0194**
Positive	ref		ref	
***Received TB vaccination***				
Yes	0.577 (0.283–1.178)	0.131	0.611 (0.290–1.288)	0.1953
No	ref		ref	
***SARS-CoV-2 seroprevalence***				
Yes	1.104 (0.532–2.293)	0.7904	1.156 (0.549–2.436)	0.7025
No	ref		ref	
***Household size***	1.127 (1.006–1.261)	0.0386	1.100 (0.974–1.242)	0.1244

Note: TB denotes tuberculosis.

**Table 5:** Logistic regression analysis of factors associated with COVID-19-related symptoms within the **pandemic cancer** populations.

Cancer Population	Unadjusted Analysis	*p*-Value	Adjusted Analysis	*p*-Value
***Age***	1.01 (0.996–1.024)	0.1683	1.012 (0.997–1.027)	0.1199
***Sex***				
Female	1.129 (0.713–1.788)	0.6057	1.021 (0.633–1.647)	0.9324
Male	ref		ref	
***HIV status***				
Negative	1.049 (0.678–1.621)	0.8312	0.924 (0.568–1.503)	0.7498
Positive	ref		ref	
***Received TB vaccination***				
Yes	**2.602 (1.198–5.652)**	**0.0157**	**2.511 (1.114–5.661)**	**0.0264**
No	ref		ref	
***SARS-CoV-2 seroprevalence***				
Yes	0.755 (0.488–1.166)	0.2052	0.702 (0.449–1.098)	0.1213
No	ref		ref	
***Household size***	0.975 (0.905–1.050)	0.4973	0.977 (0.905–1.054)	0.5478

Note: TB denotes tuberculosis.

**Table 6:** Logistic regression analysis of factors associated with COVID-19-related symptoms among SARS-CoV-2 seropositive within the **pandemic potential blood donors**.

Potential Blood Donors Population	Unadjusted Analysis OR (95% CI)	*p*-Value	Adjusted Analysis OR (95% CI)	*p*-Value
***Age***	1.018 (0.997–1.039)	0.0999	1.007 (0.984–1.031)	0.5272
***Sex***				
Female	**2.571 (1.452–4.551)**	**0.0012**	**1.950 (1.062–3.581)**	**0.0312**
Male	ref		ref	
***HIV status***				
Negative	**0.295 (0.134–0.646)**	**0.0023**	**0.305 (0.134–0.694)**	**0.0047**
Positive	ref		ref	
***Received TB vaccination***				
Yes	**0.441 (0.212–0.918)**	**0.0287**	0.482 (0.223–1.042)	0.0635
No	ref		ref	
***Household size***	1.088 (0.958–1.237)	0.1931	1.050 (0.914–1.208)	0.49

Note: TB denotes tuberculosis.

**Table 7:** Logistic regression analysis of factors associated with COVID-19-related symptoms among SARS-CoV-2 seropositive within the **pandemic cancer** populations.

Cancer Population	Unadjusted Analysis	*p*-Value	Adjusted Analysis	*p*-Value
***Age***	**1.026 (1.007–1.045)**	**0.006**	**1.029 (1.010–1.049)**	**0.0034**
***Sex***				
Female	1.213 (0.685–2.150)	0.508	0.957 (0.524–1.746)	0.8849
Male	ref		ref	
***HIV status***				
Negative	1.032 (0.607–1.756)	0.907	0.885 (0.493–1.591)	0.6837
Positive	ref		ref	
***Received TB vaccination***				
Yes	1.982 (0.799–4.916)	0.1401	1.894 (0.722–4.969)	0.1944
No	ref		ref	
***Household size***	0.945 (0.860–1.038)	0.2356	0.943 (0.854–1.042)	0.251

Note: TB denotes tuberculosis.

## Data Availability

All data underlying this article are available in the article.

## Supplementary Materials

The following supporting information can be downloaded at: https://www.mdpi.com/article/10.3390/jcm11154428/s1.
