# Supplementary material for: Lower SARS-CoV-2 Seroprevalence among Cancer Patients in Sub-Saharan Africa
Source: J Clin Med. 2022 Jul 29;11(15):4428. doi: 10.3390/jcm11154428 (PMC9369079; doi:10.3390/jcm11154428)
Supplement: Supplementary file 1 [file jcm-11-04428-s001.zip › jcm-1813222-supplementary.pdf]

Supplementary Table S1: Distribution of different cancers in pandemic population.

**Cancer type**	Sample size	Percentage
Cervix	204	42.95
Breast	63	13.26
Kaposi sarcoma	38	8
Head and Neck	37	7.79
Colorectal	32	6.74
Esophagus	29	6.11
Skin	10	2.11
Prostate	7	1.47
Vulva	7	1.47
Lung	6	1.26
Others	42	8.84

Note: ‘Others’ includes 25 different types of cancers such as penile cancer, tongue cancer, gastrointestinal stroma cancer, hematological malignancies (Lymphoma, Leukemia, and Multiple myeloma), just to mention a few.

Supplementary Table S2a: Multivariable logistic regression analysis of the relationship between SARS-CoV-2 total seropositivity **(Spike + Nucleocapsid)** and study participants category (cancer versus non-cancer) within the Pandemic populations.

**Characteristic**	Unadjusted Analysis OR (95% CI)	P-value	Adjusted Analysis OR (95% CI)	P-value
Participants category				
Cancer	0.460 (0.352-0.600)	0.0001	0.535 (0.380-0.753)	0.0003
Non-cancer	ref		ref	
Age	0.988 (0.980-0.997)	0.007	0.999 (0.988-1.009)	0.7869
Sex				
Female	1.257 (0.965-1.637)	0.09	0.923 (0.690-1.234)	0.5868
Male	ref		ref	
HIV status				
Negative	1.635 (1.168-2.288)	0.0041	1.266 (0.877-1.829)	0.2081
Positive	ref		ref	
Received TB vaccination				
Yes	1.358 (0.917-2.013)	0.1269	1.376 (0.918-2.063)	0.1225
No	ref		ref	
COVID-19 related symptoms				
Yes	0.636 (0.448-0.903)	0.0113	0.817 (0.564-1.184)	0.2852
No	ref		ref	
Household size	0.940 (0.893-0.989)	0.017	0.972 (0.920-1.027)	0.3088

Note: TB denotes tuberculosis.

Supplementary Table S2b: Multivariable logistic regression analysis of the relationship between SARS-CoV-2 seropositivity **(Spike)** and study participants category (cancer versus non-cancer) within the Pandemic populations.

**Characteristic**	Unadjusted Analysis OR (95% CI)	P-value	Adjusted Analysis OR (95% CI)	P-value
Participants category				
Cancer	0.350 (0.275-0.445)	0.0001	0.431 (0.318-0.585)	0.0001
Non-cancer	ref		ref	
Age	0.981 (0.973-0.988)	0.0001	0.994 (0.984-1.003)	0.171
Sex				
Female	1.473 (1.165-1.861)	0.001	0.997 (0.768-1.295)	0.984
Male	ref		ref	
HIV status				
Negative	1.954 (1.443-2.645)	1E-04	1.377 (0.987-1.921)	0.06
Positive	ref		ref	
Received TB vaccination				
Yes	1.383 (0.970-1.972)	0.073	1.354 (0.934-1.964)	0.11
No	ref		ref	
COVID-19 related symptoms				
Yes	0.629 (0.457-0.864)	0.004	0.923 (0.656-1.299)	0.646
No	ref		ref	
Household size	0.937 (0.895-0.981)	0.006	0.984 (0.936-1.035)	0.532

Note: TB denotes tuberculosis.

Supplementary Table S2c: Multivariable logistic regression analysis of the relationship between SARS-CoV-2 seropositivity **(Nucleocapsid)** and study participants category (cancer versus non-cancer) within the Pandemic populations.

Characteristic	Unadjusted Analysis OR (95% CI)	P-value	Adjusted Analysis OR (95% CI)	P-value
Participants category				
Cancer	0.627 (0.490-0.803)	0.0002	0.721 (0.526-0.989)	0.042
Non-cancer	ref		ref	
Age	0.995 (0.987-1.003)	0.2142	1.001 (0.992-1.011)	0.763
Sex				
Female	1.084 (0.852-1.379)	0.5123	0.872 (0.669-1.135)	0.307
Male	ref		ref	
HIV status				
Negative	1.554 (1.134-2.130)	0.0061	1.394 (0.989-1.966)	0.058
Positive	ref		ref	
Received TB vaccination				
Yes	1.173 (0.808-1.704)	0.4024	1.213 (0.828-1.777)	0.321
No	ref		ref	
COVID-19 related symptoms				
Yes	0.724 (0.520-1.008)	0.056	0.831 (0.586-1.180)	0.301
No	ref		ref	
Household size	0.939 (0.895-0.984)	0.0091	0.951 (0.904-1.000)	0.05

Note: TB denotes tuberculosis.

Supplementary Table S3a: Multivariable logistic regression analysis of the relationship between SARS CoV-2 total seropositivity **(Spike + Nucleocapsid)** and study participants (cancer versus non-cancer) within the Prepandemic populations.

**Characteristic**	Unadjusted Analysis OR (95% CI)	P-value	Adjusted Analysis OR (95% CI)	P-value
Participants category				
Cancer	0.662 (0.433-1.013)	0.06	0.518 (0.255-1.052)	0.069
Non-cancer	ref		ref	
Age	1.005 (0.988-1.023)	0.57	1.015 (0.996-1.034)	0.127
Sex				
Female	0.973 (0.665-1.423)	0.886	0.776 (0.512-1.175)	0.231
Male	ref		ref	
HIV status				
Negative	1.400 (0.894-2.192)	0.141	0.951 (0.477-1.897)	0.887
Positive	ref		ref	

Supplementary Table S3b: Multivariable logistic regression analysis of the relationship between SARS CoV-2 seropositivity **(Spike)** and study participants (cancer versus non-cancer) within the Prepandemic populations.

Characteristic	Unadjusted Analysis OR (95% CI)	P-value	Adjusted Analysis OR (95% CI)	P-value
Participants category				
Cancer	0.095 (0.006-1.617)	0.1037	0.168 (0.009-3.308)	0.241
Non-cancer	ref		ref	
Age	0.986 (0.932-1.043)	0.624	1.010 (0.956-1.067)	0.717
Sex				
Female	1.285 (0.375-4.407)	0.6901	0.693 (0.211-2.281)	0.5464
Male	ref		ref	
HIV status				
Negative	8.193 (0.482-139.396)	0.141	1.930 (0.094-39.564)	0.6696
Positive	ref		ref	

Supplementary Table S3c: Multivariable logistic regression analysis of the relationship between SARS CoV-2 seropositivity **(Nucleocapsid)** and study participants (cancer versus non-cancer) within the Prepandemic populations.

Characteristic	Unadjusted Analysis OR (95% CI)	P-value	Adjusted Analysis OR (95% CI)	P-value
Participants category				
Cancer	0.685 (0.447-1.048)	0.081	0.541 (0.265-1.102)	0.091
Non-cancer	ref		ref	
Age	1.005 (0.987-1.022)	0.607	1.014 (0.995-1.033)	0.163
Sex				
Female	0.973 (0.663-1.430)	0.891	0.789 (0.519-1.201)	0.27
Male	ref		ref	
HIV status				
Negative	1.357 (0.866-2.127)	0.183	0.944 (0.472-1.889)	0.871
Positive	ref		ref	
